# Inhaled high molecular weight hyaluronan ameliorates respiratory failure in acute COPD exacerbation: a pilot study

**DOI:** 10.1186/s12931-020-01610-x

**Published:** 2021-02-01

**Authors:** Flavia Galdi, Claudio Pedone, Christopher A. McGee, Margaret George, Annette B. Rice, Shah S. Hussain, Kadambari Vijaykumar, Evan R. Boitet, Guillermo J. Tearney, John A. McGrath, Audrey R. Brown, Steven M. Rowe, Raffaele A. Incalzi, Stavros Garantziotis

**Affiliations:** 1 Division of Geriatrics, Department of Medicine, Campus Bio-Medico University and Teaching Hospital, Rome, Italy; 2 Division of Intramural Research, National Institute of Environmental Health Sciences, 111 TW Alexander Dr, Research Triangle Park, NC 27709 USA; 3 Department of Medicine and the Gregory Fleming James Cystic Fibrosis Research Center, University of Alabama in Birmingham Medical Center, Birmingham, USA; 4 Social and Scientific Systems, Durham, USA; 5 Wellman Center for Photomedicine, Massachusetts General Hospital, Boston, USA; 6 Department of Dermatology, Massachusetts General Hospital, Boston, USA; 7 Harvard Medical School, Boston, USA; 8 Harvard-MIT Division of Health Sciences and Technology, Cambridge, USA; 9 Department of Pathology, Massachusetts General Hospital, Boston, USA; 10 Department of Pediatrics, UAB, Birmingham, USA; 11 Department of Cell development & Integrative Biology, UAB, Birmingham, USA

## Abstract

**Background:**

Acute exacerbations of chronic obstructive pulmonary disease (AECOPD) carry significant morbidity and mortality. AECOPD treatment remains limited. High molecular weight hyaluronan (HMW-HA) is a glycosaminoglycan sugar, which is a physiological constituent of the lung extracellular matrix and has notable anti-inflammatory and hydrating properties.

**Research question:**

We hypothesized that inhaled HMW-HA will improve outcomes in AECOPD.

**Methods:**

We conducted a single center, randomized, placebo-controlled, double-blind study to investigate the effect of inhaled HMW-HA in patients with severe AECOPD necessitating non-invasive positive-pressure ventilation (NIPPV). Primary endpoint was time until liberation from NIPPV.

**Results:**

Out of 44 screened patients, 41 were included in the study (21 for placebo and 20 for HMW-HA). Patients treated with HMW-HA had significantly shorter duration of NIPPV. HMW-HA treated patients also had lower measured peak airway pressures on the ventilator and lower systemic inflammation markers after liberation from NIPPV. In vitro testing showed that HMW-HA significantly improved mucociliary transport in air–liquid interface cultures of primary bronchial cells from COPD patients and healthy primary cells exposed to cigarette smoke extract.

**Interpretation:**

Inhaled HMW-HA shortens the duration of respiratory failure and need for non-invasive ventilation in patients with AECOPD. Beneficial effects of HMW-HA on mucociliary clearance and inflammation may account for some of the effects (NCT02674880, www.clinicaltrials.gov).

## Background

The morbidity and mortality associated with chronic obstructive pulmonary disease (COPD) is increasing worldwide. The World Health Organization predicts that COPD will become the third leading cause of death worldwide by 2030 [1]. COPD remains the third leading cause of death in the United States [2] and by 2020 it was projected to lead to a staggering $49 billion cost [3]. Acute exacerbations of COPD (AECOPD) may occur in > 90% of COPD patients [4] and often necessitate hospitalization [5], which is the most important cost driver in COPD [6–8]. Among hospitalized patients, morbidity and health care costs are associated with severe AECOPD, which leads to need for high-level and prolonged care [9]. Treatment for AECOPD remains limited, including bronchodilators, non-specific anti-inflammatory corticosteroids, and antibiotics, and novel, effective anti-inflammatory agents are needed.

High molecular weight hyaluronan (HMW-HA) is a naturally occurring sugar, that is abundant in the extracellular matrix, including in the lung. HMW-HA has several properties that make it an attractive candidate as a therapeutic in AECOPD: HMW-HA is a very hydrophilic molecule [10], and has been used as an airway hydrating agent for several years [11], which may improve mucociliary transport in COPD. HMW-HA ameliorates airway hyperresponsiveness in murine models [12–14] and humans [15], has potent anti-inflammatory properties and ameliorates lung epithelial injury [16–18]. We therefore hypothesized that inhaled HMW-HA may be beneficial in AECOPD, especially in patients with impeding respiratory failure. This randomized, placebo-controlled, double-blind study included patients with severe AECOPD, necessitating bi-level non-invasive positive pressure ventilation (NIPPV). We reasoned that inhaled HMWHA may assist in improving lung function and therefore shorten the need for NIPPV in these patients.

## Methods

### Study design

This was a parallel-arm, randomized, placebo-controlled, double-blinded, single-center pilot study of patients admitted to the Intermediate Care Section, Geriatric Unit, Campus Bio-Medico, University of Rome, between March 2016 and July 2019. Included were adult patients with known history of physician-diagnosed COPD and acute exacerbation requiring NIPPV, as evidenced by respiratory distress (new-onset moderate to severe dyspnea and use of accessory breathing muscles) and hypercapnic respiratory failure (partial pressure of CO_2_ > 45 mm Hg in arterial blood gas analysis, new or increased compared to baseline, when a baseline value was available). Exclusion criteria were respiratory arrest or need for immediate intubation, overt pneumonia on chest X-ray, significant non-COPD contributing factors to respiratory failure (e.g. congestive heart failure), contraindications to NIPPV (upper airway obstruction, facial trauma) or inability to consent or cooperate with NIPPV. After informed consent, patients were randomized 1:1 to receive study treatment, either HMW-HA or identical-looking placebo. The HMW-HA preparation was Yabro^®^ (5 ml of saline containing 0.3% hyaluronic acid sodium salt, Institute Biochimique SA, Lugano, Switzerland). Yabro^®^ and placebo were generous donations of the manufacturer, who had the key to the randomization schema. Patients were treated with NIPPV using a Hamilton C1 ventilator and medical therapy according to current guidelines (inhaled β2 agonists, inhaled anticholinergic agents, inhaled and systemic corticosteroids, antibiotics) [19]. NIPPV was temporarily interrupted for the duration of nebulization. Patients received Yabro^®^ or placebo via a non-ultrasonic jet nebulizer (Vincal^®^, which guarantees appropriate nebulizer output as instructed in the Yabro^®^ package leaflet) twice daily, until they were liberated from NIPPV or until NIPPV failure, defined as need for orotracheal intubation due to difficulty in managing airway secretions, worsening arterial blood gas parameters, or cardiorespiratory arrest. The decision to liberate from NIPPV was reached as follows: for patients not on home/chronic NIPPV, NIPPV was discontinued when an improvement of pH and/or a pCO2 < 55 was reached. For patients using home/chronic NIPPV (4/21 in the placebo group and 3/20 in the Yabro^®^ group), NIPPV was reset to home settings when an improvement of pH and/or pCO_2_ was reached, and this was considered to be equivalent to “liberation from NIPPV” status. The study was co-sponsored by the National Institute of Environmental Health Sciences, National Institutes of Health, USA, and the Campus Bio-Medico, University of Rome, Italy. The manufacturer had no role in the conduct or analysis of this study. Serum was collected on admission and after liberation from NIPPV and stored at − 80 °C until analyzed.

### Outcomes

The primary outcome was duration until liberation from NIPPV or NIPPV failure. Secondary outcomes were: (a) respiratory physiology parameters (ventilator-recorded peak pressures as an indicator of airway resistance; partial arterial O_2_ and pCO_2_ pressures on arterial blood gas analysis); and (b) markers of systemic inflammation associated with AECOPD (C-reactive protein; Interleukin 6 [IL-6]; Interleukin 8 [IL-8]; C-X-C Motif Chemokine Ligand 10 [CXCL10]) [20–24]. Adverse events were recorded regardless of whether they were deemed to be related to treatments.

### Analysis

Cumulative hours on NIPPV were plotted on GraphPad Prism, version 8, and analyzed using the log-rank Mantel-Cox test. NIPPV days and hospital length-of-stay values were analyzed for normality using GraphPad Prism software and comparisons were performed either using the Student’s t-test or Mann–Whitney test according to normality. Categorical variables were analyzed using Fisher’s exact test. For inflammation markers, values for the 4 analytes (CRP, CXCL10, IL-6, and IL-8) were ln-transformed to more closely approximate normality. Data were cast in “long” format with 8 records per subject: 4 reactants × 2 times (pre- and post-treatment) and analyzed using SAS 9.4 Proc Mixed, regressing the ln(reactant values) on treatment group (HMW-HA vs. placebo), timing (pre vs. post), and reactant type, with timing and reactant type as repeated measures, and subject ID entered to capture the subject effect. In initial models we included the 3-way interaction of group × timing × reactant, plus all possible 2-way interactions and main effects. We tested this 3-way model with three different Kronecker product covariance structures [25], which allow for the modeling of two repeated measures by combining two covariance matrices. We chose a combination of two unstructured covariance matrices as providing the best fit, based on the Akaike Information Criterion. We ran additional models, removing the 3-way and non-significant 2-way interactions and ran separate mixed models for the 2 groups, to obtain least-square means for pre- and post- times, to assess significant change for any inflammation markers.

### Air–liquid interface culture

To gain novel mechanistic insights into the effect of HMW-HA effects in COPD, we analyzed its effect on mucociliary clearance, since airway clearance is important for both chronic and acute COPD management [26]. Primary human bronchial epithelial (HBE) cells were isolated from lung explants from COPD patients and no lung disease control donors, expanded in submerged culture for one or two passages in Bronchial epithelial growth medium (BEGM, Lonza, Walksville, MD), and then seeded on Transwell membranes (Corning, New York, NY) as described previously [27, 28]. Cells were grown at air liquid interface until terminally differentiated for 4–5 weeks using PneumaCult ALI media (StemCell technology). The UAB Institutional Review Board approved use of primary human cells.

### Cigarette smoke extract preparation and exposure to cells with treatments

Cigarette smoke extract were generated using filtered cigarettes (3R4F: University of Kentucky, USA) and absorbed on Dimethyl Sulphoxide (DMSO), then sterile filtered before using in cell culture and stored at − 80 °C for further use [29, 30]. Differentiated epithelial cells were treated with 1% CSE or vehicle control apically and/or apical treatment by HA (0.3%) or vehicle control (1 µL) for 24 h.

### µOCT imaging and analysis

To assess the functional microanatomy in cell culture, µOCT imaging was performed before and 24 h after treatment conditions, as described [31–33]. Airway surface liquid (ASL), periciliary layer (PCL) depth, ciliary beat frequency (CBF), and mucociliary transport rates (MCT) were measured using Image J (NIH) and customized MatLab scripts available from UAB or MGH. MCT was determined tracking native mucus particles over time. For each measure, at least 5 random locations (or trackable particles) per region of interest (ROI) were obtained and averaged, and for each monolayer, 4 systematically obtained ROIs were averaged to obtain a single value per monolayer. Data were analyzed by two or three operators blinded to treatment assignment and presented as single average per monolayer for which quantitative statistics were performed. Statistical analysis used repeated measure ANOVA with Sidak’s post hoc testing.

## Results

### Patient population

**Fig. 1:**
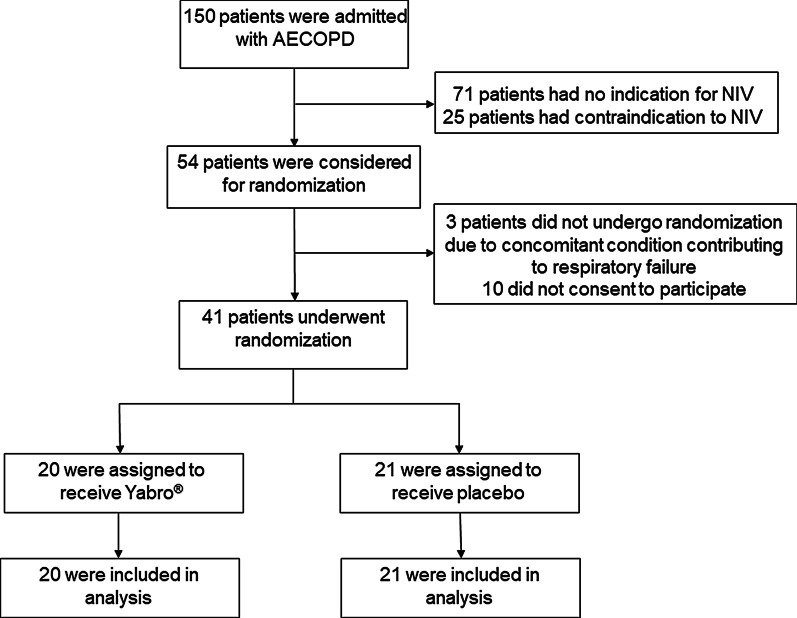
Study schema. Of 150 patients admitted with AECOPD during the study period, 51 were eligible to participate and 41 consented to be included in the study

The physicians at Campus Biomedico have developed a home care monitoring system based on wearable sensors which allows detection of AECOPD in early stages [34]. Accordingly, most patients with AECOPD are treated at home. Selected patients are admitted to a day hospital for lab analyses and imaging, as needed, to optimize their home care. Thus, only patients who failed to improve after these interventions were hospitalized. In the period from March 2016 to July 2019, 150 patients were admitted with AECOPD. Of these, 71 had no indication for NIPPV, 25 had contraindications to NIPPV, and 10 refused to consent to the study. Of the remaining 44 who were screened for eligibility to the study, 41 were included, while 3 were excluded because they were deemed to have a concomitant condition (congestive heart failure) that contributed significantly to their presentation (Fig. 1). Twenty patients received HMW-HA and 21 received placebo. Baseline characteristics of the two groups were similar for demographics, medical history, admission lung physiology and laboratory parameters (Table 1). There was no difference in the rate of adverse events in the two treatment groups: One patient in the HMW-HA group suffered a fatal upper gastrointestinal tract hemorrhage (from pre-existing peptic ulcer disease), one patient in the placebo group suffered a hydropneumothorax, and were thus withdrawn for further study. Three more patients withdrew consent (N = 2 for placebo, N = 1 for HMW-HA), and were censored as “not liberated from NIPPV” for intention-to-treat analyses. Another patient in the placebo group progressed to respiratory failure necessitating mechanical ventilation and ultimately died. Complications after HMW-HA or placebo inhalation were specifically tracked, and none were reported.

**Table 1:** Baseline characteristics of patients included in this study

	Placebo	HMW-HA (Yabro®)	p-value
Demographics			
N	21	20	
Age	74.2 ± 10	77.4 ± 8.5	0.85
Male (%)	35	35	1.00
Medical History (% of patients with history)			
Home O_2_ therapy	57	55	1.00
COPD exacerbation requiring hospitalization in past year	43	30	0.52
Home NIV use	19	15	1.00
Coronary Artery Disease	57	60	0.85
Neurovascular Disease	9.5	25	0.19
Chronic Kidney Disease	19	25	0.64
Diabetes	29	40	0.44
Hypertension	62	85	0.09
Past Pneumonia	38	25	0.37
Peptic Ulcer Disease	0	15	0.06
IADL Score	4.9 ± 2.6	4.7 ± 2.0	0.83
Admission Vital Signs			
Mean arterial pressure (mmHg)	89 ± 9	87 ± 12	0.70
Heart Rate (per minute)	75 ± 10	81 ± 11	0.10
Respiratory Rate (per minute)	27 ± 5	27 ± 4	0.73
Temperature (°C)	36.0 ± 0.4	35.9 ± 0.5	0.47
Body Mass Index	26.9 ± 8.6	24.7 ± 5.7	0.50
Admission Respiratory Parameters			
FiO2 (%)	27 ± 8	29 ± 7	0.38
PaO2 (mmHg)	62 ± 12	60 ± 15	0.59
PaCO2 (mmHg)	66 ± 13	63 ± 9	0.40
pH	7.34 ± 0.04	7.34 ± 0.04	0.95
IPAP	16.1 ± 3.4	16.3 ± 3.8	0.70
PEEP	6.1 ± 1.1	5.5 ± 0.7	0.10
Admission Laboratory Values			
Na (mEq/L)	138 ± 3	139 ± 3	0.96
K (mEq/L)	4.1 ± 0.4	4.0 ± 0.5	0.21
Creatinine (mg/dL)	1.0 ± 0.5	1.0 ± 0.6	0.96
Hematocrit (%)	40.2 ± 6.4	39.8 ± 12.1	0.90
WBC (× 1,000 cells/µL)	10.2 ± 4.2	9.8 ± 3.9	0.74
APACHE II score	14.9 ± 2.7	14.7 ± 3.7	0.85
Positive sputum culture	4/21^#^	3/20*	1.00

Groups do not differ with regards to demographics, medical history, or admission vital signs, respiratory parameters or laboratory values (entered as average ± SD). p-values calculated by Student’s t-test or Wilcoxon (depending on normality of the data), or Fisher’s exact test for categorical variables. IADL = Score for Instrumental Activities of Daily Living [58]. FiO_2_ = fraction of inhaled oxygen. PaO_2_ = Partial pressure of oxygen in arterial blood. PaCO_2_ = Partial pressure of carbon dioxide in arterial blood. IPAP = inspiratory positive airway pressure. PEEP = positive end-expiratory pressure. WBC = white blood cell count. APACHE = Acute Physiology and Chronic Health Evaluation [59]

^#^ One patient each with methicillin-resistant *S. aureus*, *K. pneumoniae*, *P. aeruginosa*, and *E. coli* + *P. aeruginosa*

^*^ One patient each with methicillin-resistant *S. aureus*, *H. influenzae*, and *E. coli* + *H. influenzae* + *P. aeruginosa*

### Duration of non-invasive ventilation

**Fig. 2:**
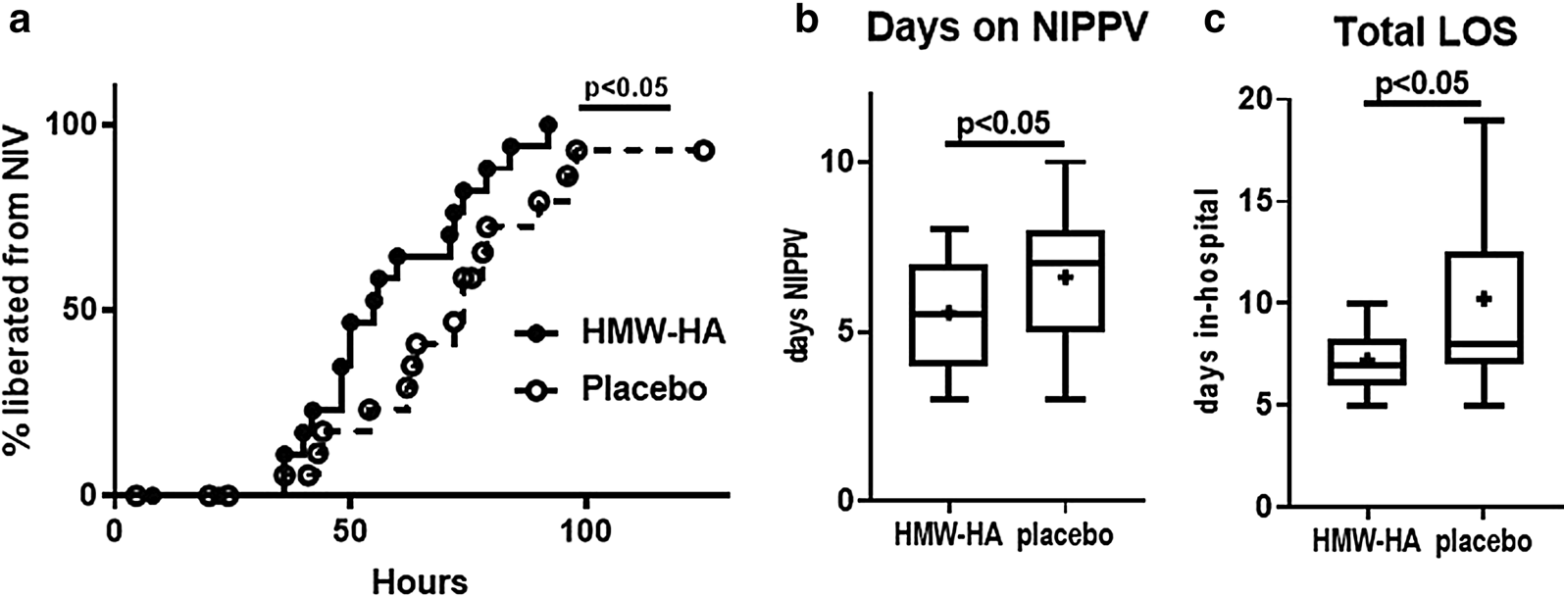
HMW-HA treatment reduces duration of NIPPV in AECOPD. **a** Kaplan–Meier curve of cumulative hours on NIPPV in intention-to-treat analysis. HMW-HA treated patients have significantly shorter time to liberation from NIPPV N = 20 for HMW-HA patients, N = 21 for placebo patients, intention-to-treat analysis. Patients who suffered an adverse event and were withdrawn from the study (N = 1 from each group), or withdrew consent (N = 2 for placebo, N = 1 for HMW-HA), were censored as “not liberated from NIPPV” for this analysis. One patient in the placebo group failed NIPPV, was orotracheally intubated and was censored as “not liberated from NIPPV” at 125 h (point of intubation). **b** Duration of NIPPV measured in hospital days. HMW-HA treated patients were liberated from NIPPV on average 1 day earlier compared to placebo-treated patients. N = 18 for both groups (2 patients in placebo group and one in HMW-HA group withdrew consent, and one patient each suffered an adverse event and was withdrawn from the study by the study physician). **c** Total length of stay. HMW-HA treated patients were discharged from the hospital on average 2 days earlier. N = 18 for both groups (explanation as in **b**)

The primary outcome for this study was cumulative duration on NIPPV. We found that patients treated with HMW-HA were liberated from NIPPV significantly faster than placebo-treated patients (Fig. 2a). The effect was clinically significant: on average, patients treated with HMW-HA were liberated from NIPPV more than a day earlier than placebo-treated patients: average days on NIPPV: 5.2 ± 0.4 for HMW-HA vs. 6.4 ± 0.5 days for placebo, (average ± SEM), 5 ± 3 vs. 7 ± 3 (median ± IQR), p = 0.037, t-test) (Fig. 2b). This was associated with overall shortened total length of stay (LOS) in the hospital: average ± SEM 7.2 ± 0.3 for HMW-HA vs. 10.2 ± 1.3 days for placebo, median ± IQR 7 ± 2.25 vs. 8 ± 5.5, p = 0.039 by Mann–Whitney (Fig. 2c). These data suggest that HMW-HA shortened the duration of acute respiratory failure, need for NIPPV and consequently hospital LOS in these patients.

### Respiratory function effects

**Fig. 3:**
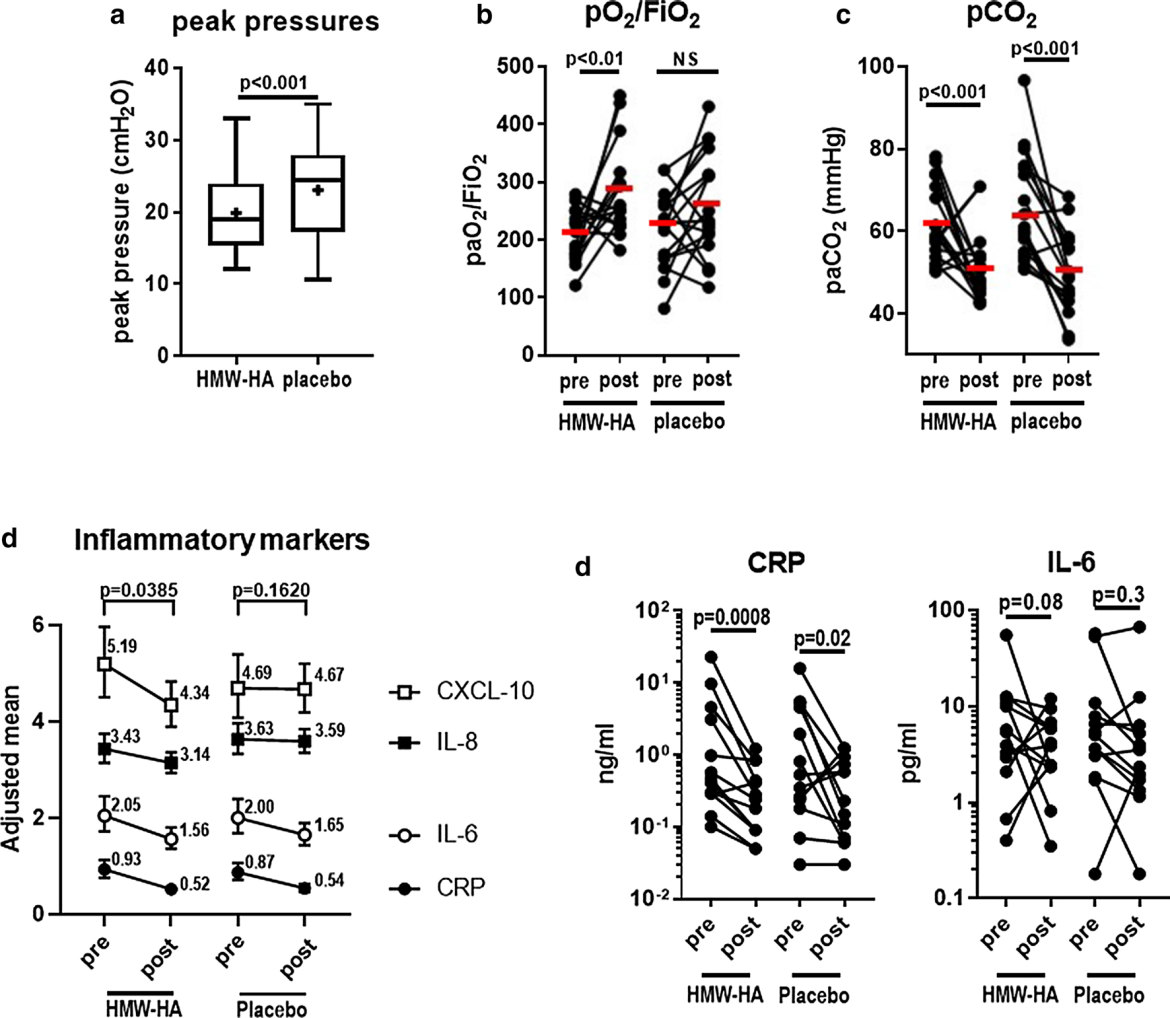
Improved lung function and serum markers of inflammation in HMW-HA treated patients. **a** HMW-HA-treated patients have lower peak pressures, as recorded by the NIV apparatus, throughout NIVVP. **b** paO_2_/FiO_2_ ratio improves significantly in HMW-HA patients. **c** paCO_2_ improves significantly in both groups. N = 17 per group. **d** Joint analysis of 4 serum inflammatory markers shows a significant improvement in HMW-HA treated patients, but not in placebo patients. **e** Among the 2 inflammatory markers (CRP and IL-6) that decreased with treatment in both groups in **a**, more robust improvement can be seen in HMW-HA patients compared to placebo-treated patients. N = 13 per group

Patients treated with HMW-HA had significantly lower peak pressures reported by the ventilator software (Fig. 3a). Arterial blood gas values improved, as expected, with treatment. Interestingly, the pO_2_/FiO_2_ ratio improved significantly only in the HMW-HA treated group, comparing admission and day of liberation from NIPPV (from 210 ± 46 to 284 ± 78, p = 0.011) and not in the placebo group (from 218 ± 66 to 261 ± 91, p = 0.085) (Fig. 3b). Notably, because samples were obtained at the day of liberation from NIPPV and not on a fixed day after initiation of NIPPV, these samples were obtained on average a day earlier in the HMW-HA group. pCO_2_ values improved significantly in both groups (Fig. 3c), possibly reflecting the fact that pCO_2_ values were part of the clinical decision-making process for liberation from NIPPV. In aggregate, these results suggest improved lung function in association with HMW-HA treatment.

### Systemic markers of inflammation

For logistical reasons, we only evaluated serum markers of inflammation on admission and on the day of liberation from NIPPV. Unfortunately, some samples were destroyed inadvertently while in transit from Italy to the US lab for analysis, therefore only 13 samples per group could be analyzed. When we tested 3-way interaction of treatment effect with timing (pre-post) and inflammation marker, we did not observe a significant difference between HMW-HA and placebo. In both groups, acute phase inflammation marker values generally decreased over time, reflecting the general health improvement for patients. Interestingly however, HMW-HA-treated patients had a more pronounced response in inflammation markers than placebo-treated patients. When we ran separate models for each group, in placebo patients the timing-effect interaction lost significance (p = 0.162), while in HMW-HA-treated patients the timing-effect interaction remained significant (p = 0.039) (Fig. 3d). When we analyzed acute phase inflammation markers separately, there was a general trend for HMW-HA-treated patients to have more robust effects (e.g. CRP in placebo patients decreased from a natural logarithm-transformed value of -0.142 to -0.615 (p = 0.02) while the HMWHA-treated group decreased from -0.072 to -0.651 (p = 0.0008). Similar effects could be observed in the other 3 cytokines and chemokines measured (Fig. 3d, e). Together, these results suggest that HMW-HA may have beneficial effects in systemic inflammation associated with AECOPD.

### Air–liquid interface cultures

**Fig. 4:**
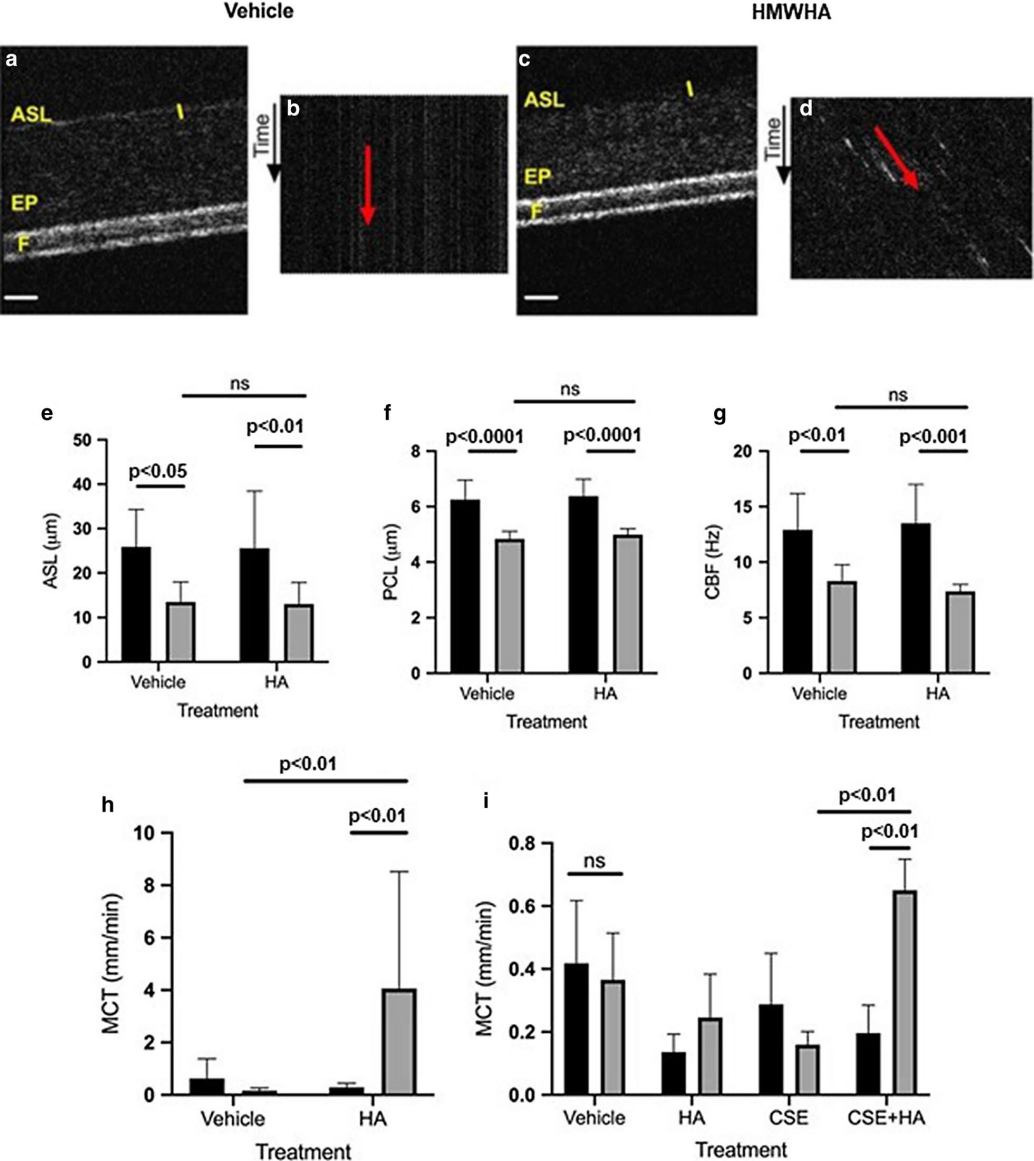
Mucociliary transport is augmented by HMW-HA. **a**–**h** Terminally-differentiated primary HBE monolayers derived from patients with COPD were treated with HMW-HA (0.3%) or an equal volume of vehicle control, then assessed at baseline and after 24 h by µOCT. Representative cross-sectional µOCT images at 24 h after vehicle (**a**) or HA (**c**) treatment. EP = epithelial monolayer; ASL depth denoted with yellow bar; F = transwell filter; white scale bar = 20 µm. Streak diagram showing time-dependent reprocessing for vehicle (**b**) or HA (**d**) are also shown. The angle of the streaks, as denoted by the red arrow, correspond to MCT rates. Quantitation of airway surface liquid (ASL) depth (**e**), periciliary layer (PCL) depth (**f**), ciliary beat frequency (CBF, **g**), and mucociliary transport (MCT) rate (**h**). N = 8 monolayers/condition derived from 2 different donors. **i** MCT from similar study conducted from HBE cells derived from a lung-healthy donor treated with CSE (1%, apically) and vehicle control or HMW-HA (0.3% apically) for 24 h. N = 12 monolayers/condition derived from 3 different donors. *ns* not significant

To investigate the mechanism of HMW-HA-induced improvement in lung function and liberation from NIPPV, we conducted an in vitro study using primary human bronchial epithelial (HBE) cells derived from patients with COPD. In other experiments we studied HBE cells from healthy non-smokers exposed to cigarette smoke extract (CSE). We evaluated functional microanatomy using µOCT before and after addition of HMW-HA in comparison to vehicle control (Fig. 4). Representative cross-sectional images (Fig. 4a, c) and quantitative data (Fig. 4e-g) showed no meaningful treatment-related differences on ASL or PCL depth or CBF; however, a prominent effect on mucociliary transport (MCT) with HA treatment was observed on cells from COPD patients (Fig. 4b, d, h). Similarly, cells from healthy non-smokers exposed to CSE exhibited improved MCT with HMW-HA treatment (Fig. 4i). These results suggest that patients with COPD or active smokers may derive a beneficial effect from HMW-HA through improved mucociliary transport function.

## Discussion

In this work we demonstrate that treatment with inhaled HMW-HA shortens the duration of acute respiratory failure and is associated with improved lung function and systemic inflammation in patients with AECOPD. Mechanistically, we show that HMW-HA improves mucociliary transport in airway epithelia that exhibit COPD-like manifestations. Our study shows for the first time the therapeutic potential of an extracellular matrix molecule in acute exacerbation of human lung disease.

HMW-HA had a clinically meaningful salutary effect in the primary outcome, duration of NIPPV. These results will need to be confirmed in larger studies but would suggest an important role of HMW-HA in the treatment of AECOPD, since they demonstrate added benefit in patients who were already receiving state-of-the-art AECOPD therapy. Duration of intensive care is independently associated with in-hospital mortality in AECOPD [35] and is also a strong driver of inpatient costs [36]. HMW-HA-treated patients also had a significantly shortened overall inpatient LOS. Thus, HMW-HA is of potential benefit to patients and health systems.

HMW-HA is found in high concentrations in the lung matrix under physiological conditions [37] and is detectable on the luminal surface of airway epithelia [38]. However, during acute inflammation HMW-HA is degraded to smaller fragments [12, 39, 40], which have strong pro-infammatory properties [41]. Accumulation of HA fragments appears to be a common thread in many chronic lung diseases [42], including COPD [43, 44]. This process appears to be proportional to disease activity; HA levels in sputum and blood have been associated with COPD severity, degree of inflammation and patient survival [45, 46]. Emerging evidence suggests that imbalance between declining HMW-HA levels, and increasing smaller fragments of HA may contribute to chronic airway disease pathogenesis [47]. This has led to the hypothesis, that exogenous supplementation of HMW-HA may restore HA homeostasis in favor of undegraded molecules, inhibit inflammation and loss of lung function and ameliorate COPD progression [48]. Aerosolized HA was found to reverse emphysema induced by neutrophil elastase [49] and cigarette smoke [50] in animal models, although the precise mechanism remains unclear. Our work adds to the existing evidence for therapeutic utility of HA in COPD, by demonstrating beneficial effects in AECOPD, and a connection to improved mucus clearance.

HMW-HA possesses several beneficial properties which make it an attractive candidate as an exogenously administered treatment option in inflammatory lung disease. HMW-HA is a naturally occurring sugar [37], and therefore is expected to be well tolerated. Indeed, our study did not demonstrate any apparent adverse effects with Yabro^®^ inhalation. This is not unexpected; inhaled HMW-HA has been used routinely, together with hypertonic saline, in cystic fibrosis patients with no reported side effects; rather it improves tolerability and decrease the need for bronchodilators in these patients [51, 52]. In addition, HMW-HA is very hydrophilic [10]. This property may contribute to the improvement in airway pressures observed in HMW-HA-treated patients. Interestingly, the hydrating effect did not seem to contribute significantly in the improvement in MCT seen in our in vitro study. MCT is dictated primarily by ciliary function and mucus rheology. While short fragments of HA have been shown to increase CBF [38], HMW-HA does not have such an effect, as we showed in this study as well. Furthermore, the ASL and PCL dimensions did not change with HMW-HA, arguing against a significant effect on the hydration status of the mucus layer. However, HMW-HA (but interestingly, not low-MW HA) changes the nanostructure of mucin, possibly through entanglement with mucin proteins [53] and could thus improve the functional rheology of the mucociliary layer [47] and promote MCT through this mechanism. In support of this interpretation, recent studies have shown that normalizing mucin structure can improve MCT in cystic fibrosis airways [54].

HMW-HA also has potent anti-inflammatory and pro-homeostatic properties. In murine models, HMW-HA ameliorates acute allergic and non-allergic inflammation [14, 55, 56] and lung epithelial injury [16, 18]. E*x vivo*, HMW-HA inhibited LPS-induced TNFα release by whole blood from COPD patients [57]. Thus, HMW-HA may ameliorate airway inflammation in AECOPD as well. We were not able to collect sputum in order to quantify lung inflammation markers. However, serum inflammatory markers seemed to respond favorably to inhaled HMW-HA. While no single biomarker has yet been shown to be highly predictive of outcomes in AECOPD, we analyzed markers that have been associated with AECOPD [20–24]. In aggregate, our analysis suggested that HMW-HA treatment resulted in greater improvement in serum inflammatory markers over time, compared to placebo (Fig. 3). In addition, it should be noted that, since the serum samples were collected at the end of NIPPV, HMW-HA-treated patients were on average sampled a day earlier than placebo-treated patients (because they were liberated from NIPPV a day earlier on average, Fig. 2). Thus, HMW-HA treatment effects may have been underestimated in our study. Studies with a higher number of patients and more frequent sampling strategies will help confirm this observed trend.

Another mechanism of improved lung function by HMW-HA administration may be reduction of bronchial constriction. Several studies have shown that HMW-HA ameliorates inflammation-associated airway hyperresponsiveness in murine models [12–14] and in humans [15]. The observed lower peak airway pressures in HMW-HA patients, observed in our study, would suggest that airway resistance was reduced in these patients. We did not observe any difference in documented tidal volumes throughout the study duration (not shown), but this could be due to changes in ventilatory support dictated by the patients’ clinical status.

There are several limitations to this pilot study. Due to the small number of patients and the single-center nature of the study, its results would certainly need to be validated and replicated in larger studies. This study stretched over 2–3 years, thus introducing a potential variability in treatment approaches. However, this was at least partially counteracted by the fact that only 2 dedicated physicians led the treatment of recruited patients throughout the study duration. We unfortunately did not have background information regarding lung function or baseline blood gas values in these patients, and therefore we cannot fully comment on the severity of pre-existing COPD. However, the two groups were well-matched with regard to clinical parameters, i.e. home O_2_ and NIPPV use, thus we would expect that there were no significant differences in lung function either. Also, as described in “Methods” section, our COPD patients were managed with a telemedicine approach which significantly reduces the need for hospitalization [34]. Thus, only relatively treatment-resistant cases were admitted and shuttled into the randomization schema. This may also explain why our documented pH values at admission were only borderline acidemic, despite significant elevation of pCO_2_: these were not patients with hyperacute exacerbation, but rather progressively declining patients who failed to respond to outpatient management, and therefore had time to at least partially compensate their acid–base status. Finally, these patients represent cases of community- “acquired” COPD exacerbation, and therefore it is not clear if HMWHA benefits also apply to cases of deterioration in the inpatient setting, or in the case of patients that need invasive ventilation.

## Conclusion

Inhaled HMWHA may be beneficial in severe AECOPD, by improving inflammation and lung function, and reducing the need for ventilatory support. Our study supports the use of a novel class of treatment, i.e. matrix biologics, in acute and chronic lung disease. Additional studies should confirm our findings with higher number of patients in additional clinical sites and explore the use of HMWHA in chronic COPD, both to reduce COPD progression and to decrease exacerbations which are the major driver of morbidity, mortality and cost in this devastating disease.

## Data Availability

The datasets analyzed during the current study are available from the corresponding author on reasonable request.

## Abbreviations

AECOPD: Acute exacerbation of chronic obstructive pulmonary disease
APACHE: Acute Physiology and Chronic Health Evaluation
ASL: Airway surface liquid
CBF: Ciliary beat frequency
CRP: C-reactive protein
CXCL10: C-X-C Motif Chemokine Ligand 10
FiO_2_: Fraction of inhaled oxygen
HMW-HA: High molecular weight hyaluronic acid (hyaluronan)
IADL: Score for Instrumental Activities of Daily Living
IL-6: Interleukin 6
IL-8: Interleukin 8
IPAP: Inspiratory positive airway pressure
MCT: Mucociliary transport rate
μOCT: One-micron optical coherence tomography
NIPPV: Non-invasive positive pressure ventilation
PaCO_2_: Partial pressure of carbon dioxide in arterial blood
PaO_2_: Partial pressure of oxygen in arterial blood
PCL: Periciliary layer depth
PEEP: Positive end-expiratory pressure
WBC: White blood cell count
